# Post-treatment alpha fetoprotein and platelets predict hepatocellular carcinoma development in dual-infected hepatitis B and C patients after eradication of hepatitis C

**DOI:** 10.18632/oncotarget.24219

**Published:** 2018-01-13

**Authors:** Ming-Lun Yeh, Ching-I Huang, Chung-Feng Huang, Meng-Hsuan Hsieh, Ming-Yen Hsieh, Zu-Yau Lin, Shinn-Cherng Chen, Jee-Fu Huang, Po-Lin Kuo, Hsing-Tao Kuo, Chia-Yen Dai, Ming-Lung Yu, Wan-Long Chuang

**Affiliations:** ^1^ Graduate Institute of Medicine, College of Medicine, Kaohsiung Medical University, Kaohsiung, Taiwan; ^2^ Hepatobiliary Division, Department of Internal Medicine, Kaohsiung Medical University Hospital, Kaohsiung, Taiwan; ^3^ School of Medicine, College of Medicine, Kaohsiung Medical University, Kaohsiung, Taiwan; ^4^ Lipid Science and Aging Research Center, Kaohsiung Medical University, Kaohsiung, Taiwan; ^5^ Department of Occupational and Environmental Medicine, Kaohsiung Medical University Hospital, Kaohsiung, Taiwan; ^6^ Division of Hepatogastroenterology, Department of Internal Medicine, Chi-Mei Medical Center, Tainan, Taiwan; ^7^ Department of Senior Citizen Service Management Chia Nan University of Pharmacy and Science, Tainan, Taiwan; ^8^ Institute of Biomedical Sciences, National Sun Yat-Sen University, Kaohsiung, Taiwan

**Keywords:** hepatocellular carcinoma, incidence, hepatitis B virus, hepatitis C virus, dual infection

## Abstract

We investigated the long-term risk of hepatocellular carcinoma (HCC) in dual-infected hepatitis B and C patients after eradication of hepatitis C virus (HCV). A total of 164 (62% male, median age, 50.5 years) hepatitis B and C dual-infected patients who achieved HCV sustained virological response were recruited. Half the patients were HCV genotype 1 with a median viral load of 5.5 log10 IU/mL, and 22.6%had an HBV DNA level ≥ 2000 IU/mL before therapy. HCC developed in 14 patients (8.5%), with an annual incidence of 1.38% per person-year. The 3-year, 5-year, 10-year, and 15-year cumulative probabilities were 2.5%, 5.1%, 12.6%, and 22.7%, respectively. Six months after treatment, a Cox regression hazard analysis revealed platelet level (HR: 0.98, 95% CI: 0.957–0.999, *P* = 0.038) and AFP level (HR: 1.20, 95% CI: 1.031–1.400, *P* = 0.019) to be independent factors in HCC. A higher 10-year cumulative risk of HCC was detected in patients with 6-month post-treatment AFP levels > 5.0 ng/mL and platelet levels < 130 x1000/µL (54.9%), compared to patients with neither (8.6%). Although the risk of HCC is low, surveillance of HCC is encouraged in dual-infected patients after eradication of HCV. Post-treatment AFP and platelet levels predict HCC development.

## INTRODUCTION

A dual infection with hepatitis B virus (HBV) and hepatitis C virus (HCV) is not uncommon in the Asia-Pacific region, a high endemic area for both HBV and HCV [1]. Previous data revealed that an estimated 2% to 10% of patients with HCV infection might be co-infected with HBV [1–4]. Dual infection with the two viruses indicates patients who are at a high risk for disease aggravation and progression compared with patients with mono-infection [5, 6].

Hepatocellular carcinoma (HCC) is the leading cause of mortality in patients with chronic liver disease. Whether the risk of HCC is higher in dual-infected patients has been studied. However, the results are controversial. In the meta-analysis by Cho et al. [7], 59 participants were enrolled, including those with HBV/HCV dual-infection and HBV or HCV mono-infection. The investigators concluded that the risk of HCC in HBV/HCV dual-infection patients is not greater than the risk of HCC in HBV or HCV mono-infection patients. A study that investigated the clinical outcomes of HBV co-infection in a United States cohort reported that patients with documented HBV viremia were at a higher risk for HCC compared with HCV mono-infected patients [8]. However, the absence of HBV replication correlated with clinical outcomes similar to those of mono-infected patients. A study from Taiwan concluded that HCV co-infection is an independent risk factor for HCC [9].

A reduced risk of HCC was confirmed in HCV mono-infected patients who had been treated with interferon (IFN) therapy and who had achieved a sustained virological response (SVR) [10, 11]. However, the effect of IFN therapy was limited in patients who received pretreatment HCC after curative therapy [12]. For HBV/HCV dual-infected patients, IFN therapy demonstrated a similar HCV SVR rate compared with HCV mono-infected patients [13–15]. The prior study also demonstrated that HCV SVR reduced the risk of HCC after IFN therapy in a median follow-up period of 4.6 years [16]. However, the long-term risk and associated predictor of HCC development in HBV/HCV dual-infected patients after HCV eradication remains uncertain. In the present study, we investigated the long-term risk of HCC development in patients with HBV/HCV dual-infection after HCV eradication. We also investigated whether any factors predict which patients are at a high risk for HCC development.

## RESULTS

Table 1 shows the demographics of all patients with and without HCC development. Of all the patients, 61.6% were males. The median age was 50.5 years. Almost 13% of the patients had liver cirrhosis before therapy. Half of the patients were HCV genotype 1, with a median viral load of 5.5 log_10_ IU/mL. Ninety percent of patients had the interleukin-28B (IL-28B) rs8099917 TT genotype. Before and 6months after treatment, 22.6% and 20.2% of the patients had an HBV DNA level greater than or equal to 2000 IU/mL, respectively. Most of the patients (87.8%) received pegylated IFN therapy. Ten patients received HBV oral antiviral treatment after HCV SVR, and HCC developed in three of those patients.

**Table 1:** Demographics of all the HBV/HCV dual-infected patients with HCV SVR after IFN therapy

	All (*n* = 164)
Pretreatment	
Male gender	101 (61.6)
Age, years	50.5 (45.0, 57.0)
Body mass index, kg/m^2^	24.9 (22.8, 27.3)
Liver cirrhosis	21 (12.8)
Platelet, 10^3^/mm^3^	162.0 (125.0, 194.0)
AST, U/L	65.0 (42.0, 94.0)
ALT, U/L	88.5 (55.0, 154.0)
γ-GT, U/L	36.0 (23.0, 65.0)
HCV genotype 1	85 (51.8)
HCV RNA, log_10_ IU/mL	5.5 (4.5, 6.0)
IL28B rs8099917 TT genotype	114/127 (89.8)
HBV DNA ≥ 2000 IU/mL	30/133 (22.6)
Pegylated IFN	144 (87.8)
6 months after treatment	
Platelet, 10^3^/mm^3^	168.0 (133.0, 195.0)
ALT, U/L	25.0 (19.0, 33.0)
γ-GT, U/L	22.0 (16.0, 38.0)
AFP, ng/mL	3.4 (3.0, 5.0)
HBV DNA ≥ 2000 IU/mL	26/129 (20.2)

AST, aspartate aminotransferase; ALT, alanine aminotransferase; γ-GT, gamma-glutamyltransferase; HCV, hepatitis C virus; IL28B, interleukin 28B; HBeAg, hepatitis B e antigen; HBV, hepatitis B virus; IFN, interferon; AFP, alpha fetoprotein

In the univariate analysis, patients in whom HCC developed had an older pretreatment age, a higher rate of liver cirrhosis, a lower platelet level, a lower HCV RNA level and an HBV DNA level ≥ 2000 IU/mL, a lower 6-month post-treatment platelet level, and a higher gamma-glutamyltransferase (γ-GT) level. However, in the multivariate analysis, the only significant difference was in the 6-month post-treatment platelet level (OR: 0.97, 95% CI: 0.955–0.995, *P* = 0.013) of patients with and without HCC development (Table 2).

**Table 2:** Comparison between HBV/HCV dual-infected patients with and without HCC development after HCV eradication

	HCC	Non-HCC				
	(*n* = 14)	(*n* = 150)	*p*	OR	95% CI	*p*
Pretreatment						
Male gender	10 (71.4)	91 (60.7)	0.570			
Age, years	55.5 (49.8, 61.0)	50.0 (43.0, 56.3)	0.046	1.03	0.936–1.141	0.516
Body mass index, kg/m^2^	26.0 (22.5, 28.8)	24.8 (22.8, 27.2)	0.396			
Liver cirrhosis	7 (50.0)	14 (9.3)	< 0.001	0.87	0.116–6.615	0.897
Platelet, 10^3^/mm^3^	104.5 (71.0, 145.5)	164.0 (134.0, 198.0)	< 0.001			
AST, U/L	69.0 (47.5, 81.3)	65.0 (41.5, 94.5)	0.824			
ALT, U/L	95.5 (54.0, 143.8)	88.5 (55.0, 158.5)	0.809			
γ-GT, U/L	58.5 (25.8, 103.8)	36.0 (22.0, 62.0)	0.113			
HCV genotype 1	8 (57.1)	77 (51.3)	0.783			
HCV RNA, log_10_ IU/mL	4.9 (3.2, 5.5)	5.6 (4.6, 6.1)	0.026	0.89	0.429–1.842	0.752
IL28B rs8099917 TT genotype	10/10 (100)	104/117 (88.9)	0.597			
HBV DNA ≥ 2000 IU/mL	8/13 (61.5)	22/120 (18.3)	0.002	2.98	0.573–15.455	0.195
Pegylated IFN	10 (71.4)	134 (89.3)	0.072			
6 months after treatment						
Platelet, 10^3^/mm^3^	108.0 (66.5, 144.3)	171.0 (139.5, 201.5)	< 0.001	0.97	0.955– 0.995	0.013
ALT, U/L	29.0 (24.0, 35.3)	25.0 (19.0, 33.0)	0.131			
γ-GT, U/L	35.9 (22.3, 79.3)	22.0 (16.0, 37.5)	0.018	1.00	0.966–1.037	0.960
AFP, ng/mL	4.0 (3.2, 8.3)	3.3 (3.0, 4.8)	0.078	1.16	0.896–1.508	0.257
HBV DNA ≥ 2000 IU/mL	4/13 (30.8)	22/116 (19.0)	0.295			

AST, aspartate aminotransferase; ALT, alanine aminotransferase; γ-GT, gamma-glutamyltransferase; HCV, hepatitis C virus; IL28B, interleukin 28B; HBeAg, hepatitis B e antigen; HBV, hepatitis B virus; IFN, interferon; AFP, alpha fetoprotein

### Cumulative probability of HCC development

After HCV was eradicated, HCC developed in14 (8.5%) of the 164 patients over 1014.1 person-years of follow-up. The annual incidence of HCC was 1.38% per person-year, and the 3-year, 5-year, 10-year, and 15-year cumulative probabilities of HCC were 2.5%, 5.1%, 12.6%, and 22.7%, respectively (Figure 2A). In patients with liver cirrhosis, the3-year, 5-year, and 10-year cumulative probabilities of HCC were 10.1%, 15.7%, and 37.5%, respectively, compared with 1.4%, 3.5%, and 7.4% in patients without liver cirrhosis (*P* < 0.001) (Figure 2B).

**Figure 1:**
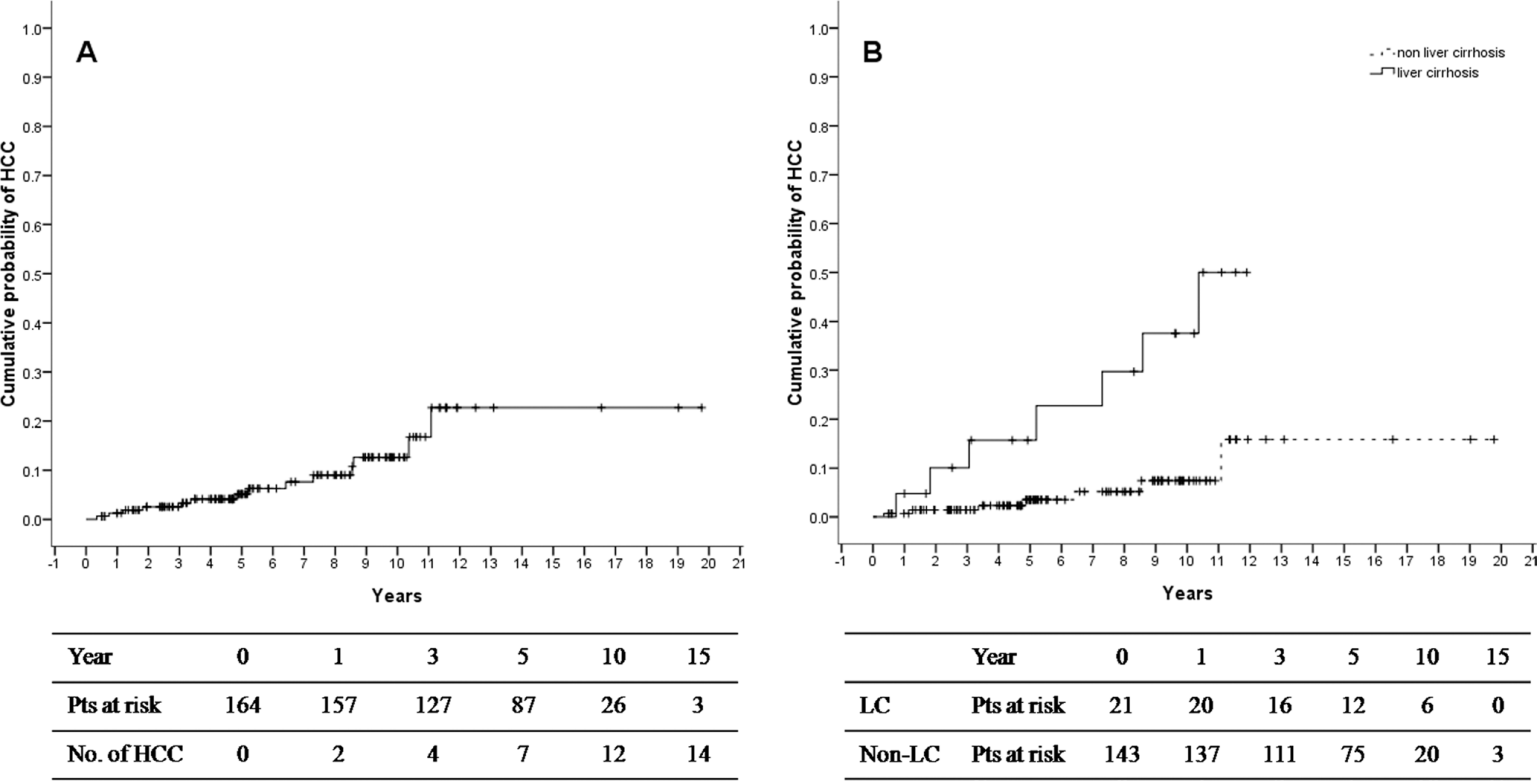
Cumulative probability of HCC development in all HBV/HCV dual-infected patients (**A**) and patients with or without liver cirrhosis (**B**) after HCV eradication.

**Figure 2:**
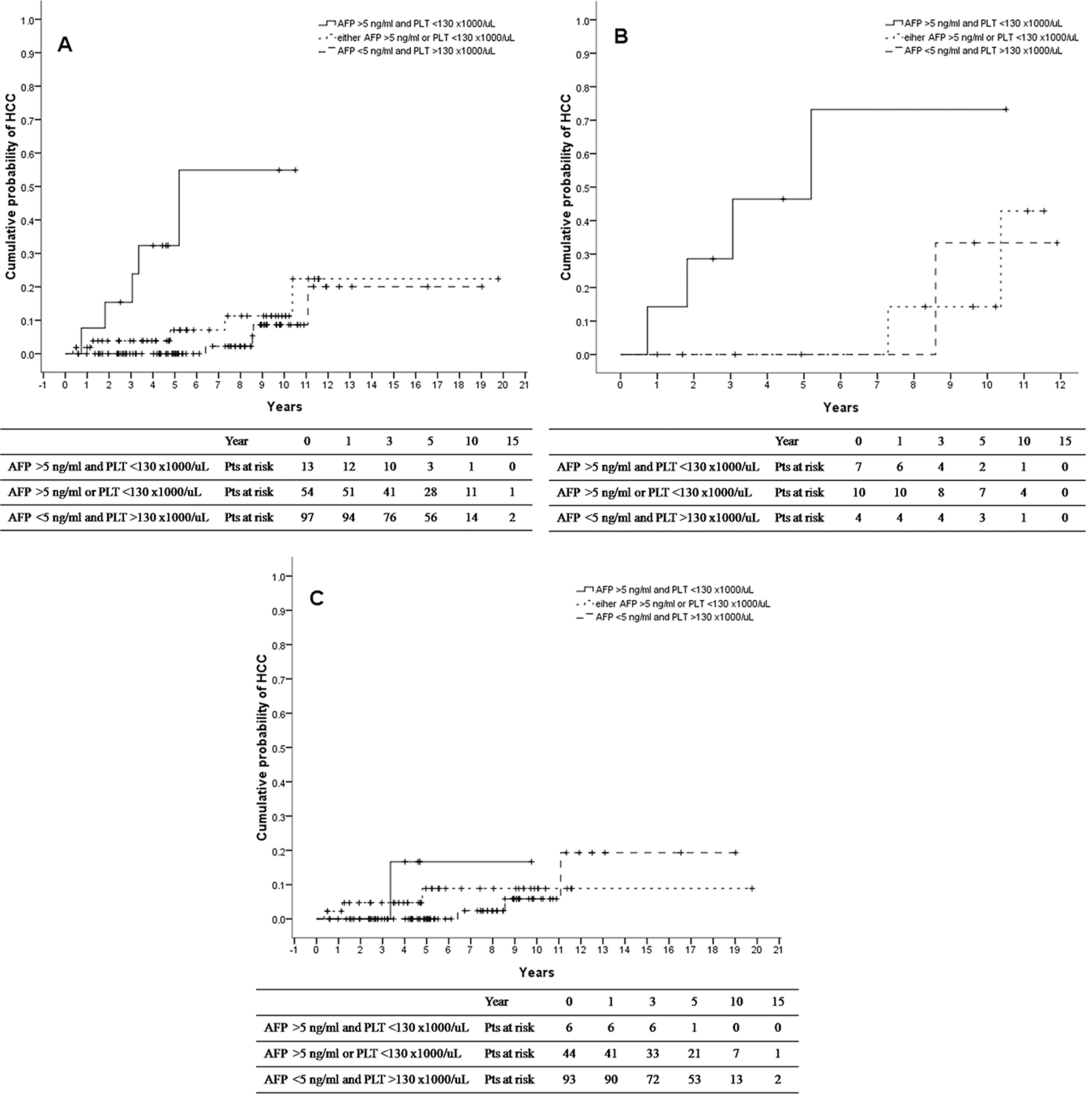
Comparison of cumulative probability of HCC development divided by 6- month post-treatment alpha-fetoprotein level 5 ng/mL and platelet 130 ×1000/µL in all patients (**A**), patients with liver cirrhosis (**B**), and patients without liver cirrhosis (**C**).

### Factors associated with HCC development

We further analyzed the factors associated with HCC development using the Cox regression hazard model. The initial analysis revealed the following: pretreatment age (HR: 1.07, 95% CI: 1.015–1.037, *P* = 0.014), liver cirrhosis (HR: 5.60, 95% CI: 1.947–16.103, *P* = 0.001), platelet level (HR: 0.98, 95% CI: 0.965–0.988, *P* < 0.001), HCV RNA level (HR: 0.63, 95% CI: 0.404–0.990, *P* = 0.045), HBV DNA ≥ 2000 IU/mL (HR: 5.98, 95% CI: 1.954–18.292, *P* = 0.002), 6-month post-treatment platelet level (HR: 0.98, 95% CI: 0.966–0.990, *P* < 0.001), γ-GT(HR:1.03, 95% CI: 1.011–1.048, *P* = 0.002), and alpha fetoprotein (AFP) level (HR:1.27, 95% CI: 1.138–1.414, *P* = 0.004). After adjusting for the pre treatment and post-treatment significant factors, we found the 6- month post-treatment platelet level (HR: 0.98, 95% CI: 0.957–0.999, *P* = 0.038) and AFP level (HR: 1.20, 95% CI: 1.031–1.400, *P* = 0.019) were independent factors correlated with HCC development (Table 3).

**Table 3:** Cox regression hazard analysis of factors associated with HCC development in the HBV/HCV dual-infected patients after HCV eradication

Parameter	Crude			Adjusted		
	HR	95% CI	*P*	HR	95% CI	*P*
Pretreatment						
Male gender	1.38	0.431–4.429	0.586			
Age, years	1.07	1.015–1.137	0.014	1.04	0.958–1.134	0.335
Body mass index, kg/m^2^	1.08	0.924–1.267	0.328			
Liver cirrhosis	5.60	1.947–16.103	0.001	0.58	0.112–2.951	0.508
Platelet, 10^3^/mm^3^	0.98	0.965–0.988	< 0.001			
AST, U/L	1.00	0.986–1.011	0.775			
ALT, U/L	1.00	0.991–1.005	0.618			
γ-GT, U/L	1.01	1.000–1.011	0.067			
HCV genotype 1	1.12	0.389–3.245	0.829			
HCV RNA, log_10_ IU/mL	0.63	0.404–0.990	0.045	1.10	0.650–1.866	0.719
IL28B rs8099917 TT genotype	23.73	0.002–347348	0.518			
HBV DNA ≥ 2000 IU/mL	5.98	1.954–18.292	0.002	1.75	0.346–8.828	0.499
Conventional IFN, n(%)	1.58	0.462–5.406	0.467			
6 months after treatment						
Platelet, 10^3^/mm^3^	0.98	0.967–0.988	<0.001	0.98	0.957–0.999	0.038
ALT, U/L	1.01	0.998–1.027	0.090			
γ-GT, U/L	1.03	1.011–1.048	0.002	1.00	0.968–1.040	0.870
AFP, ng/mL	1.27	1.138–1.414	0.004	1.20	1.03 –1.400	0.019
HBV DNA ≥ 2000 IU/mL	1.73	0.526–5.675	0.368			

AST, aspartate aminotransferase; ALT, alanine aminotransferase; γ-GT, gamma-glutamyltransferase; HCV, hepatitis C virus; IL28B, interleukin 28B; HBeAg, hepatitis B e antigen; HBV, hepatitis B virus; IFN, interferon; AFP, alpha fetoprotein

### Combined post-treatment platelet and AFP levels predict HCC development

**Figure 3:**
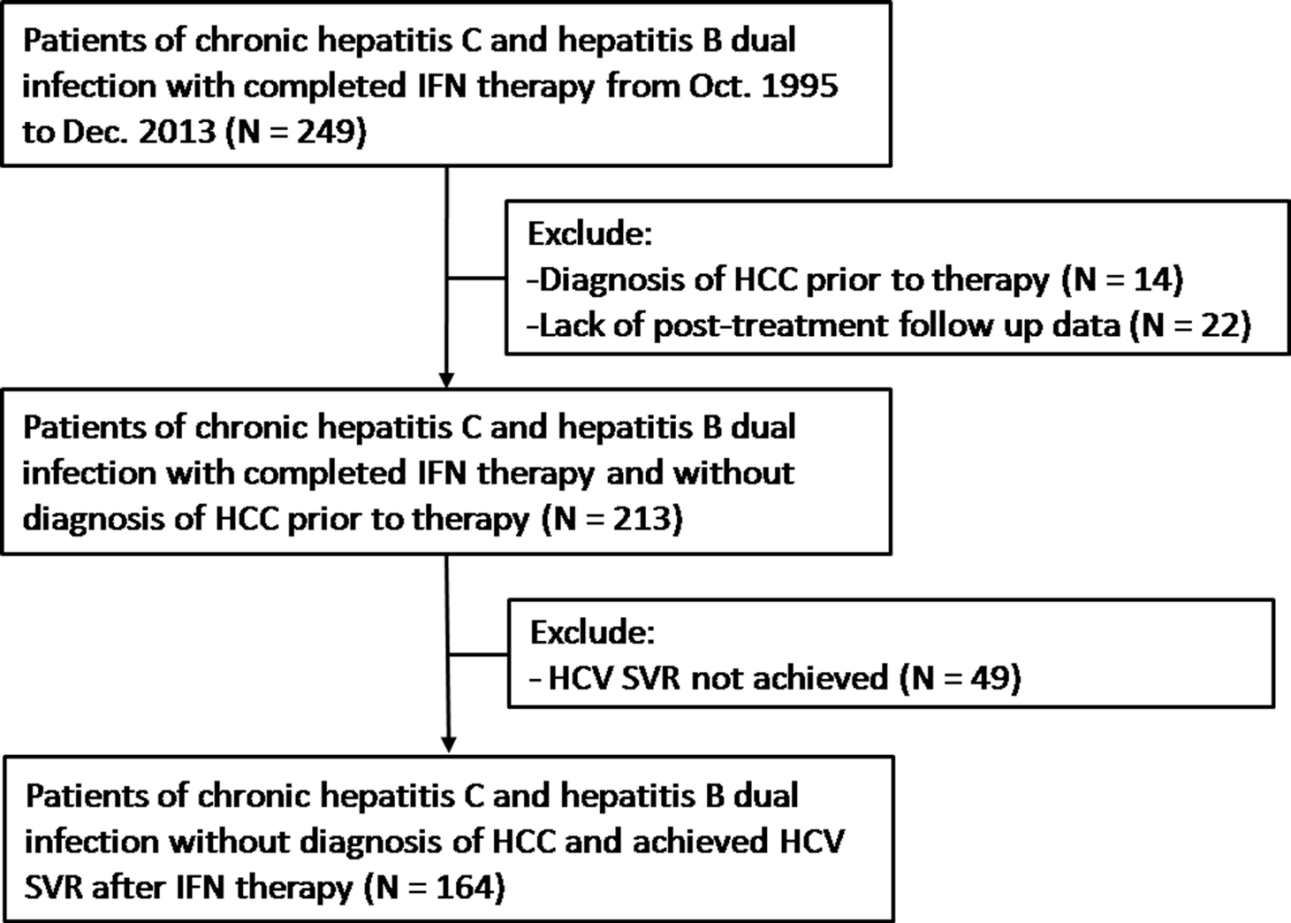
Patient allocation flowchart

By the prior analysis, we identified two independent factors that might predict HCC development. We selected the 6-month post-treatment AFP level > 5.0 ng/mL (75 percentile of AFP level) and platelet level < 130 ×1000/µL (25 percentile of platelet level) as the parameters to predict HCC development Patients with both parameters had higher risk of HCC development, compared with patients who had neither of the parameters (HR: 15.96, 95% CI 4.135– 1.598, *P* < 0.001) (Table 4). The sensitivity, specificity, positive predictive value, negative predictive value, and accuracy of the two combined parameters in predicting HCC were 35.7%, 94.6%, 38.5%, 94.0%, and 89.6%, respectively. The 3-year, 5-year, and 10-year cumulative probability of HCC were 15.4%, 32.3%, and 54.9%, respectively, in patients with both parameters, comparedwith0%, 0%, and 8.6% in patients who had neither of the parameters (Figure 3A). In patients with liver cirrhosis who had both parameters, the 3-year, 5-year, and 10-year cumulative probabilities of HCC were 28.6%, 46.4%, and 73.2%, respectively. In contrast, HCC developed in none of the patients with one or neither of the parameters within 7 years after HCV was eradicated (Figure 3B). In patients without liver cirrhosis, no significant difference was seen between the patient groups, and the 10-year cumulative probabilities of HCC were 16.7%, 8.9%, and 5.9% in patients with both, one, and neither of the parameters, respectively (Figure 3C). HCC did not develop within 6 years in patients without either of the parameters.

**Table 4:** Relative risk of HCC development comparing patients without predictive factors, patients with either predictive factor, patients with both predictive factors, 6-month post-treatment alpha fetoprotein, and platelets

	HR	95% CI	*P*
AFP < 5 ng/mLand platelet > 130 × 1000/µL (*n* = 97)	Ref		
AFP > 5 ng/mLor platelet < 130 × 1000/µL (*n* = 54)	2.21	0.591–8.233	0.239
AFP > 5 ng/mLand platelet < 130 × 1000/µL (*n* = 13)	15.96	4.135–61.598	< 0.001

## DISCUSSION

The present study was the first study to investigate the risks and associated factors of HCC development in dual-infected patients after HCV was eradicated. We found that the incidence of HCC was low, with only 1.38% per person-year, and 3-year and 5-year cumulative probabilities of 2.5% and 5.1%, respectively. We further identified that AFP and platelet levels at 6 months after treatment were the independent predictive factors of HCC. Using the two parameters, we identified the groups of patients with higher and lower risk of HCC.

The decreased risk of HCC has been seen after IFN therapy and HCV eradication in HCV mono-infected patients. A large-scale prospective study from Japan demonstrated a significantly lower risk of HCC development (5-year cumulative incidence of 1.7%) in HCV mono-infected patients who achieve HCV SVR after pegylated IFN therapy [17]. Although a decreased risk of HCC exists after HCV eradication, HCC still develops in patients [18–20]. To identify patients who were still at risk for HCC after HCV eradication, the prior study demonstrated that higher AFP level at 24 weeks after the end of IFN therapy was the predictor of HCC incidence [21]. Another study reported that elevated AFP level 24 weeks after IFN treatment was the significant risk factor for HCC in HCV mono-infected patients, even in those who achieve HCV SVR [22]. In this study, the 5-year and 10-year cumulative incidences of HCC were 1.7% and 4.8%, respectively. Older age, male gender, lower platelet count, and higher AFP level at 24 weeks after treatment were all independent factors linked to HCC. The further time-dependent receiver operating characteristic(ROC)analysis revealed that AFP had the highest predictive power for HCC incidence after SVR. Whether the AFP and platelet level predict HCC development in HBV/HCV dual-infected patients has never been studied. The present study was the first study to report the same predictive value of AFP and platelet level at 6 months after treatment in dual-infected patients as in HCV mono-infected patients.

The prediction of HCC development in dual-infected patients has seldom been studied. In a multi-center study from Taiwan, the results revealed that the risk of HCC decreased after dual-infected patients achieved HCV SVR after IFN therapy, as in HCV mono-infected patients [16]. Another study from Taiwan investigated the factors associated with HCC in dual-infected patients. The investigators found that Pre-S deletion, A1762T/G1764A mutation, and HCV genotype 1 were the factors associated with HCC [23]. However, in that study, neither post-treatment factors nor AFP level was analyzed. In the present study, we analyzed not only pretreatment factors but also post-treatment factors that were routinely checked in clinical practice.

A major difference between HBV/HCV dual-infection and HCV mono-infection is the influence of HBV. Studies have shown that HBV DNA level correlates with HCC development in patients with HBV infection. HBV DNA level might also be a predictor of HCC in HBV/HCV dual-infected patients. However, we found that the HBV DNA level did not influence HCC incidence in dual-infected patients after HCV was eradicated. This discrepancy might be a result of the relatively small number of cases. However, HBV DNA level might, in fact, not be an important factor in HCC development in dual-infected patients. A further large-scale study is needed to clarify the issue.

One limitation of our study was the lack of HBV DNA in some of the patients. Because of the reimbursement policy, we could measure the viral load level of only one of the viruses, and usually, the HCV RNA would be measured. Therefore, not all the patients had pretreatment and post-treatment HBV DNA levels measured.

We found that patients with HBV/HCV dual infection were still at risk for HCC development, even after eradication of HCV. Surveillance of HCC was encouraged, especially for patients with a high AFP and low platelet level at 6 months after treatment.

## MATERIALS AND METHODS

### Patients

The present long-term follow-up study was conducted at one tertiary medical center and two core regional hospitals in southern Taiwan. From October 1995 to December 2013, 249 patients dually infected with HBV and HCV who received IFN-based therapy were evaluated. Of the 249 patients, 14 patients with a diagnosis of HCC before completion of IFN therapy and 22 patients who lacked post-treatment follow-up data were excluded from the present study. Of the remaining 213 patients, 49 patients who did not achieve HCV SVR after IFN therapy were excluded, and a total of 164 dual-infected patients who achieved HCV SVR were recruited. The patient allocation flowchart is shown in Figure 1. The inclusion criteria of the present study were(1) age ≥ 20 years; (2) seropositive for hepatitis B surface antigen (HBsAg), HCV antibodies (Anti-HCV), and HCV RNA for 6 months; (3) completion of IFN, either conventional or pegylated IFN therapy; (4) no diagnosis of HCC before the completion of IFN therapy. The exclusion criteria were human immunodeficiency virus infection; another liver disease except for HBV and HCV infection, such as autoimmune hepatitis, primary biliary cirrhosis, sclerosing cholangitis, Wilson disease, and α1-antitrypsin deficiency; and any other known disease that was not suitable for IFN therapy.

### Laboratory testing

HBsAg, hepatitis B e antigen, and antibody to hepatitis B e antigen were tested by use of commercially available enzyme-linked immunosorbent assay kits (Abbott Laboratories, North Chicago, IL, USA). Anti-HCV was determined by a third-generation enzyme immunoassay (Abbott Laboratories). HCV RNA was measured by use of a qualitative polymerase chain reaction assay, with a detection limit of 50 IU/mL (CobasAmplicor Hepatitis C Virus Test, Version 2.0; Roche Diagnostics, Branchburg, NJ). Serum levels of HCV RNA were quantified by the branched DNA assay, with a quantification limit of 615 IU/mL (Versant HCV RNA 3.0, Bayer, Tarrytown, NJ) if qualitative HCV RNA was seropositive. HCV genotypes were determined by use of the method described by Okamoto et al. [24]. Serum HBV DNA levels were determined by use of the CobasAmpliPrep/CobasTaqMan HBV assay, with a dynamic range of 20 IU/mL–1.7 × 10^8^IU/mL (CAP/CTM Version 2.0, Roche Diagnostics, Indianapolis, IN, USA). Stored serum was used for the HBV DNA test, if available. Liver cirrhosis was diagnosed by either histology or ultrasound diagnosis combined with evidence of portal hypertension, such as splenomegaly, esophageal, or gastric varices.

### IL-28B genotyping

On the basis of our previous studies [25–27], the IL-28B rs8099917 genotype was selected as the candidate single nucleotide polymorphism (SNP) in the current study. Genotypes of the patients were determined by the ABI TaqMan^®^ SNP genotyping assays (Applied Biosystems, Foster City, CA, USA) with the by use of pre-designed commercial genotyping assays (ABI Assay ID: C__11710096_10). PCR primers and two allelic-specific probes were designed to detect the specific SNP target. The PCR reactions were performed in 96-well microplates with ABI 7500 real-time PCR (Applied Biosystems, Foster City, USA). Allele differentiation was achieved by detection of fluorescence with System SDS Software, Version 1.2.3.

The study was conducted according to Declaration of Helsinki guidelines and Good Clinical Practice principles and was approved by the local ethics committees. Written informed consent was obtained from all patients.

### Study endpoint

The primary aim of the study was to determine the risk of HCC development in HBV/HCV dual-infected patients after eradication of HCV. The secondary aim was to determine the factors associated with HCC development.

The HCC diagnosis was made by two radiological imaging modalities that showed the typical pictures of HCC (early enhancement in the arterial phase with early wash-out in the delay portal-venous phase), one radiological imaging modality that showed the typical pictures of HCC combined with a serum alpha-fetoprotein level ≥ 400 ng/mL, or cytological/histological diagnosis of HCC. In the hospitals, the diagnostic criteria of HCC were modified from regional guidelines, and the diagnosis of HCC for each patient was reviewed/documented by the HCC expert group.

### Statistics

Continuous variables were expressed as the median (25th, 75th percentile), and categorical variables were expressed as a number (percentage). The Mann-Whitney U test was used to compare continuous variables, and chi-squared and Fisher’s exact tests were used to compare categorical variables. Binary logistic regression analysis was performed for the comparison between patients with and without HCC development. Cox proportional hazards regression models were used to identify independent factors that might relate to HCC development. The cumulative probability of HCC development was analyzed by the Kaplan-Meier actuarial curve method with the log-rank test. All tests were two-sided, and a *P*-value < 0.05 was considered to be statistically significant. All analyses were performed by the SPSS 19.0 statistical package (SPSS, Inc., Chicago, IL, USA).
